# Outcome analysis of modified lateral malleolar osteotomy for the management of talar body fractures associated with tibial pilon fractures

**DOI:** 10.3389/fsurg.2026.1843849

**Published:** 2026-07-13

**Authors:** DeXiang Zhang, Xiao Zhong, Shaobing Zhang

**Affiliations:** Ziyang Central Hospital, Ziyang, Sichuan, China

**Keywords:** ankle, combined approach, modified lateral malleolar osteotomy, talar body fracture, tibial pilon fracture

## Abstract

**Objective:**

This study aims to evaluate the clinical efficacy of treating talar body and tibial pilon fractures using a modified lateral malleolar osteotomy approach, with a focus on its advantages.

**Methods:**

Five patients who underwent a modified lateral malleolar osteotomy for talar body and tibial pilon fractures between July 2021 and June 2024 were included. According to the Sneppen classification, there were three cases of type I and two cases of type II talar body fractures. Pilon fractures were classified as type II using the Rüedi-Allgöwer system, including two cases of AO/OTA type B2 and three cases of type B3. Three fracture cases involved the right foot and two involved the left foot. Clinical outcomes were assessed by measuring the time to a positive Hawkins sign, the American Orthopaedic Foot and Ankle Society (AOFAS) ankle-hindfoot score, the visual analog scale (VAS) pain score, and the active plantar flexion and dorsiflexion range of motion (ROM) of the affected and healthy ankles at the final follow-up.

**Results:**

Intraoperatively, the anterior talofibular ligament (ATFL) was ruptured in three cases and avulsed from the lateral malleolus in one case; only one patient had an intact ATFL. Slight darkening of the incision edge occurred in one patient; however, all incisions healed primarily. All patients were followed up for 18–40 months (mean 26.6 months). Radiographs taken three months postoperatively demonstrated union at the osteotomy site in all cases. All talar fractures healed, with a mean time to positive Hawkins sign of 13.2 ± 1.8 weeks. At the final follow-up, the mean AOFAS score was (77.8 ± 10.0) points, rated as excellent in one case, good in two, and fair in two. The mean VAS score was 1.8 ± 0.8 points. Posttraumatic arthritis developed in one patient, who tolerated the associated pain. The ankle ROM of the affected side remained lower than that of the healthy side at the final follow-up.

**Conclusion:**

The modified lateral malleolar osteotomy approach for talar body and tibial pilon fractures is technically straightforward. It provides excellent exposure to the tibiotalar joint surface and fracture fragments, facilitates reliable fixation, and yields acceptable short-term outcomes that may represent a reasonable salvage result given the severity of these injuries.

## Introduction

The talus plays a pivotal role in ankle and foot biomechanics. Although talar fractures are relatively rare, they constitute the second most frequent tarsal fracture after calcaneal injuries (1). Talar body fractures represent approximately 7%–38% of all talar fractures; however, their diagnosis is often challenging, leading to a high rate of missed or delayed detection (2). Lateral talar dome fractures are Sneppen type I lesions (3). Because the lateral malleolus obscures direct visualization, achieving anatomic reduction and stable fixation is challenging. Therefore, osteotomy of the lateral malleolus is frequently required to obtain adequate exposure of the fracture site.

**Figure 1:**
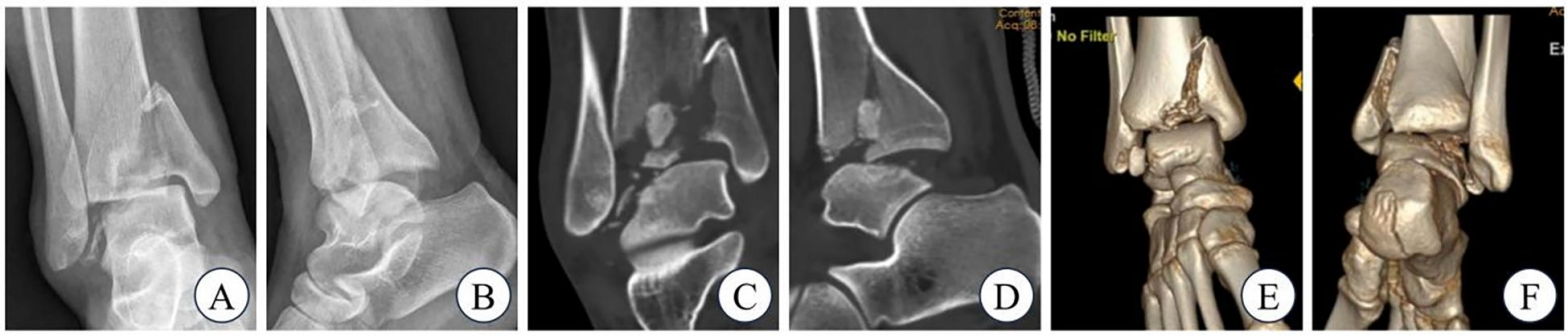
Patient: A 17-year old female was admitted 1 h after a fall from height, presenting with right ankle pain, deformity, and limited mobility. Diagnosis: 1. Right talar body fracture (Sneppen type I); 2. Right distal tibial fracture (AO/OTA 43B3, Rüedi–Allgöwer type II); 3. Right ankle subluxation; 4. Right lateral malleolar avulsion fracture. **(A,B)** Preoperative AP and lateral ankle radiographs revealing a fracture at the lateral dome of the right talus, a right tibial pilon fracture, and right ankle subluxation. **(C–F)** Preoperative CT 3D reconstruction: demonstrated a comminuted/compression fracture of the lateral talar dome, depression of the distal tibial articular surface, and the presence of a die-punch fracture fragment.

**Figure 2:**
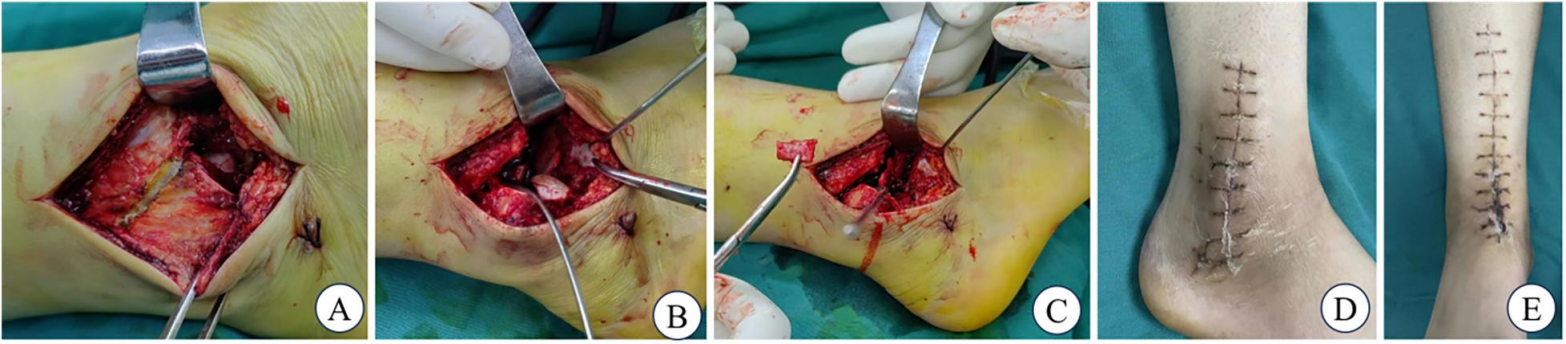
**(A)** Intraoperative exploration revealed an avulsion fracture at the fibular insertion site of the anterior talofibular ligament; the fibular osteotomy line is marked; **(B)** compression fracture of the lateral talar dome with partial cartilage avulsion; **(C)** structural bone grafting was performed using iliac bone harvest; **(D,E)** simultaneous combined anteromedial approach for open reduction and internal fixation of the pilon fracture, showing satisfactory postoperative wound healing.

**Figure 3:**
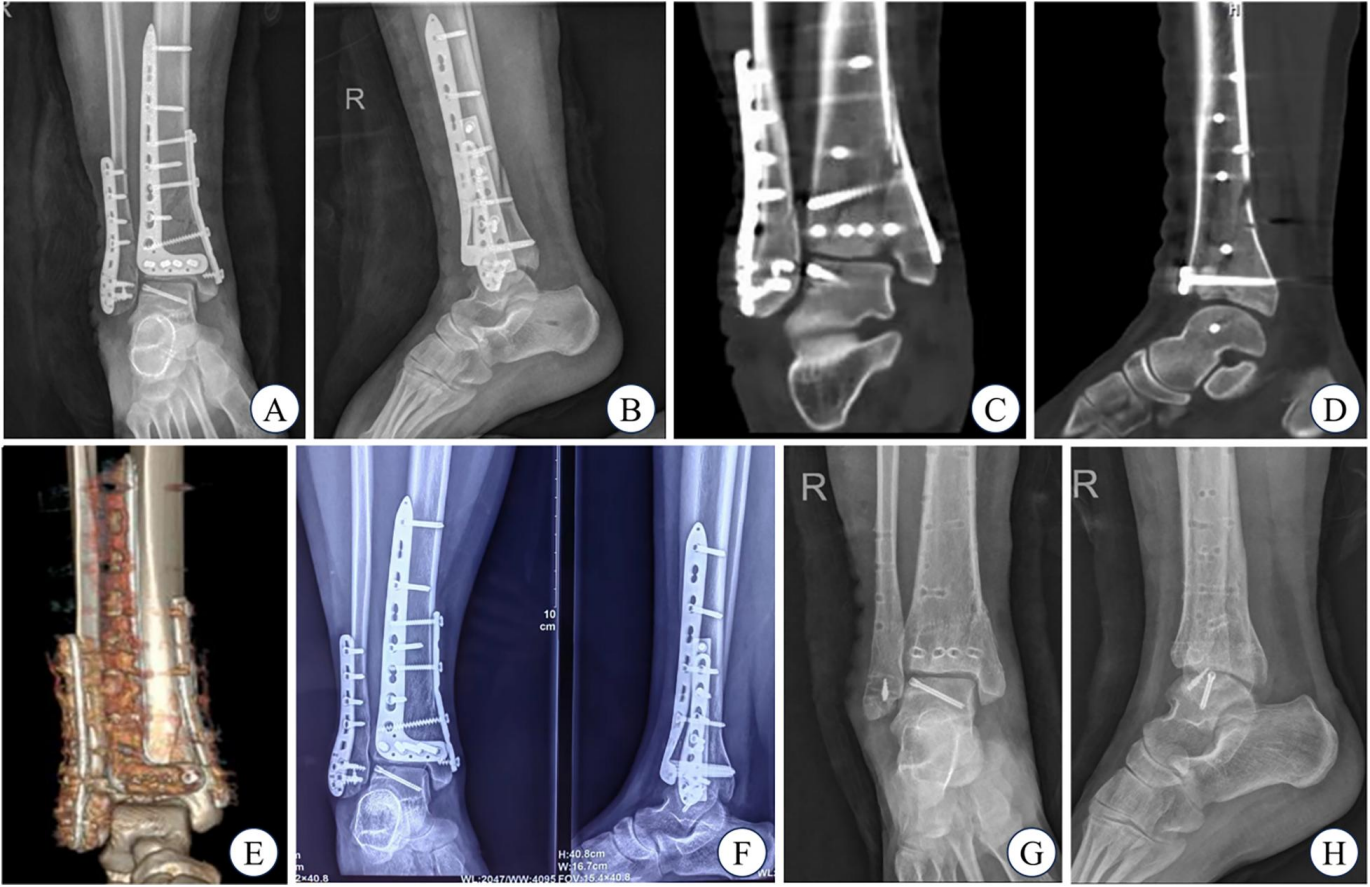
**(A–E)** Postoperative follow-up with ankle anteroposterior/lateral x-rays and CT 3D reconstruction demonstrated satisfactory fracture reduction with a well-aligned articular surface; **(F)** one-year postoperative x-rays confirmed solid fracture union without signs of traumatic arthritis; **(G,H)** internal fixation hardware was removed two years postoperatively.

The management of tibial pilon fractures remains complex and highly debated, necessitating a delicate balance among soft tissue preservation, incision planning, implant selection, and articular surface restoration (4). Both talar body and pilon fractures may progress to post-traumatic ankle arthritis, substantially compromising function and, in severe cases, requiring revision surgery, ankle arthrodesis, or even total ankle arthroplasty (5).

To permit the simultaneous visualization of the distal tibial articular surface and talar fracture fragments, this study adopted a modified lateral malleolar osteotomy approach (6) for exposure, reduction, and fixation. Short-term clinical and radiographic outcomes were encouraging, as detailed below.

## Materials and methods

### Patient cohort

Five consecutive patients who underwent modified lateral malleolar osteotomy for combined talar body and tibial pilon fractures between July 2021 and June 2024 were retrospectively reviewed. According to the Sneppen classification, three fractures were type I, and two were type II. The series comprised four females and one male, with a mean age of 25.4 years (range, 17–55 years). The right ankle was involved in three cases and the left ankle in two; all injuries resulted from high-energy falls. All pilon fractures were Rüedi–Allgöwer type II fractures. Based on the AO/OTA system, two fractures were of type 43-B2 and three were of type 43-B3. All the injuries were closed. All procedures were performed by the same senior surgeon.

### Pre-operative management

Currently, ankle dislocations are immediately reduced. Four patients underwent early skeletal traction; one patient who could not maintain ankle stability was temporarily stabilized with an external fixator. Cryotherapy (ice pack twice daily) and intravenous mannitol (250 mL, twice daily) were administered for 24 h to control swelling. Pre-operative imaging included standard anteroposterior and lateral radiography, computed tomography with three-dimensional reconstruction, and magnetic resonance imaging of the ankle to delineate articular depression, displacement, and associated injuries. The patients were instructed to perform toe-wiggling exercises and isometric contractions of the lower extremity musculature to prevent deep-vein thrombosis (DVT). Definitive surgery was scheduled once soft tissue wrinkling indicated adequate envelope recovery.

### Surgical technique

Thirty minutes before the incision, 1.0 g of cefazolin was administered intravenously. General anesthesia combined with femoral and sciatic nerve blocks was administered. A pneumatic tourniquet was inflated to 300 mmHg, and the ipsilateral buttock was elevated by 30°.

#### Lateral approach and osteotomy

An approximately 8 cm lateral ankle incision, centered over the tip of the fibula, was extended 2 cm toward the fourth metatarsal. After sharp division of the superior and inferior extensor retinacula, the distal fibula, syndesmosis, fibular attachment of the anterior talofibular ligament (ATFL), and the peroneal tendon were exposed.

The osteotomy line originated 1 cm proximal to the posterior–inferior tibiofibular ligament on the posterior fibular cortex and was directed anteroinferiorly to terminate between the anterior–inferior tibiofibular ligament and ATFL. An oscillating saw was advanced into the subchondral bone, followed by completion of the osteotomy using a thin osteotome. In patients with intact ATFLs, the ligaments were sharply transected. The distal fibular fragment was reflected posteriorly, and the ankle was positioned in plantar flexion and inversion to expose the lateral talar body and neck. The fracture debris and coagula were removed. Structural autografting with a contoured iliac crest was performed when segmental bone loss was observed. Fracture reduction was achieved and temporarily secured with guidewires. After fluoroscopic confirmation, definitive fixation was obtained with 3.0-mm Herbert headless compression screws.

#### Modified anteromedial approach

A limited anteromedial incision was made, and the tibialis anterior was retracted laterally while protecting the great saphenous vein and saphenous nerve. The distal tibial articular surface was debrided, reduced under direct vision, and temporarily stabilized using Kirschner wires. The articular reduction was simultaneously inspected through the lateral window. Definitive fixation of the distal tibia was achieved using an appropriate precontoured plate.

#### Osteotomy closure and ligament repair

The fibular osteotomy was reduced and stabilized using an anatomic locking lateral fibular plate. The fibular insertion site of the ATFL was refreshed, and a 3.5 mm metallic suture anchor was placed. With the ankle in dorsiflexion, the ATFL was reattached and augmented using the modified Broström technique. Fluoroscopy confirmed satisfactory reduction, implant positioning, joint congruity, and overall limb alignment. The wound was irrigated, a closed-suction drain was placed, and the incision was closed in layers using a uniform sterile dressing. Typical cases are shown in (Figures 1–3).

### Postoperative management and follow-up

A second dose of cefazolin (1.0 g) was administered intravenously, and the limb was elevated with intermittent ice application to minimize swelling. The drain was removed on postoperative day 1, and 4000 IU of enoxaparin was administered subcutaneously for DVT prophylaxis. Strengthening exercises for the quadriceps and triceps surae were initiated under supervision. Partial weight bearing was prohibited for the first three months. Patients were followed up at 1, 2, 3, 6, 12, and 18 months post-operatively. At each visit, functional rehabilitation was reinforced, standard radiographs were obtained to assess fracture healing, and clinical outcomes were evaluated using the American Orthopaedic Foot and Ankle Society (AOFAS) ankle–hindfoot score (7), the visual analog scale (VAS) for pain (8), and the time to appearance of Hawkins’ sign (9).

### Statistical analysis

Data were analyzed using SPSS 26.0. Given the small sample size, non-parametric methods were used throughout, and parametric assumptions were not tested. Continuous variables are presented as the median (range), with the mean ± standard deviation also reported to allow comparison with previous studies. Paired comparisons of range of motion between the affected and contralateral ankles were performed using the Wilcoxon signed-rank test. A *P*-value < 0.05 was considered statistically significant.

## Results

### General findings

Intraoperative exploration revealed anterior talofibular ligament rupture in four of the five patients; the ligament remained intact in one patient. Although superficial epidermal edge discoloration was noted in a single case, all wounds ultimately healed without the need for secondary procedures. Complete follow-up was obtained for every patient, with a duration ranging from 18 to 40 months (mean, 26.6 months) (Table 1).

**Table 1:** Basic information of patients.

Number	Gender	Age (y)	Injury site	Sneppen type	Pilon fracture classification	Whether the anterior talofibular ligament is ruptured	Follow-up time (months)	Complications	AOFAS score (points)	Results time to appearance of Hawkins’ sign (weeks)
1	female	18	left	I	AO/OTA 43B3 RÜedi－AllgÖwer II	No	24	No	83	12
2	female	42	right	II	AO/OTA 43B2 RÜedi－AllgÖwer II	Yes	28	The incision is blackened at the margin	72	16
3	female	25	right	II	AO/OTA 43B3 RÜedi－AllgÖwer II	Yes	18	No	78	12
4	male	17	right	I	AO/OTA 43B3 RÜedi－AllgÖwer II	Yes	30	No	91	12
5	female	55	left	II	AO/OTA 43B2 RÜedi－AllgÖwer II	Yes	40	Traumatic arthritis	65	14

### Clinical outcome assessment

Union. Standard radiographs taken three months after surgery showed complete healing of every lateral malleolar osteotomy and consolidation of all talar fractures. Functional score. On the AOFAS ankle–hindfoot scale, postoperative scores ranged from 65 to 91 points, with a mean of 77.8 ± 10.0. One patient was rated excellent, and two were good, yielding an excellent-to-good rate of 60%. Pain. The VAS averaged to 1.8 ± 0.8 points. Vascularity. Hawkins’ sign became evident at a mean of 13.2 ± 1.8 weeks, suggesting preserved talar blood supply in all cases. Complications. One patient developed post-traumatic ankle osteoarthritis, which was tolerable at the time of the last follow-up but may ultimately require ankle arthroplasty or arthrodesis. At the last follow-up, the affected ankle showed consistently reduced range of motion compared with the contralateral sides (Table 2).

**Table 2:** Comparison of active flexion-extension range of motion in the ankle joint: affected vs. healthy side ($\bar{x}$±s).

Item	Healthy ankle	Affected ankle	*Z* value	*P* value
Active dorsiflexion from neutral position	18.0 ± 1.4	8.4 ± 2.1	−2.02	0.063
Active plantar flexion from neutral position	42.2 ± 1.5	26.8 ± 4.8	−2.02	0.063

## Discussion

### Talar body fractures combined with tibial fractures

Concomitant fractures of the talar body and tibia are uncommon and typically result from high-energy trauma. Axial compression combined with ankle dorsiflexion and inversion/eversion is an essential mechanism for such injuries (10). All five patients in this series sustained falls from a height, which is consistent with this pathophysiology. Lahrach (11) and Moger (12) reported one case of a talar body fracture with a medial malleolar fracture, treated satisfactorily with open reduction and internal fixation (ORIF); however, Lahrach's 6-month follow-up precluded the assessment of long-term outcomes. Sanders (13) documented a rare case of a talar body fracture with open lateral malleolar and closed medial malleolar fractures, in which the distal lateral malleolar fragment was displaced and impacted posteromedially into the talus. The initial management included debridement, lateral malleolar reduction with Kirschner wire fixation, and ORIF of the talus and medial malleolus. Although fracture union without talar osteonecrosis was observed at six months, talar collapse necessitated arthrodesis 14 months postoperatively. Hamilton (14) and Githens (15) emphasized the complexity of talar fractures owing to their intricate anatomy, extensive articular cartilage coverage, and tenuous vascular supply. Concomitant tibial fractures also increase the risk of post-traumatic osteoarthritis. Nevertheless, timely ORIF, based on the fracture patterns and soft-tissue status, remains imperative. The current literature on talar body fractures with tibial pilon fractures remains sparse and poses significant management challenges.

### Limitations of existing surgical approaches for talar body fractures

Selection of the surgical approach is critical for adequate exposure and anatomical reduction. While lateral malleolar osteotomy remains the gold standard for lateral talar body fractures, existing techniques exhibit notable limitations: Liu (16) employed an inverted V-shaped osteotomy in 26 cases of lateral talar body fractures, achieving excellent/good outcomes in 23 cases. However, limited distal bone stock and challenging fragment mobilization compromise fixation and increase the risk of nonunion. Zheng (17) utilized a Y-shaped osteotomy in 12 talar body fractures, with seven patients achieving favorable outcomes. This approach preserved the anterior inferior tibiofibular ligament (AITFL) and anterior talofibular ligament (ATFL); however, the small osteotomy fragments at the ATFL insertion complicated the fixation. Zhao (18) treated 22 complex talar fracture-dislocations using a supramalleolar osteotomy (81.8% excellent/good rate). This technique requires AITFL/ATFL release and posterior malleolar reflection, necessitating syndesmotic screw fixation and ATFL repair with suture anchors, followed by screw removal at three months. Cellier (19) and Li Xiucun's team (20) reported satisfactory outcomes with arthroscopically assisted ORIF for talar body fractures. Although minimally invasive, arthroscopy entails a steep learning curve and is contraindicated in Sneppen type V fractures and in cases with concomitant tibial/calcaneal injuries; therefore, novel surgical approaches for talar body fractures are urgently needed.

### Advantages and technical considerations of the modified lateral malleolar osteotomy

Li (6) pioneered the use of preexisting fracture lines in malunited supination–external rotation ankle fractures to perform lateral malleolar osteotomies to address malunited posterior malleolar fractures. This technique involves the transection of ATFL and posterior reflection of the distal osteotomized fragment, thereby achieving optimal exposure of the articular surface of the tibiotalar joint. The patient had a satisfactory clinical outcome. Yan et al. (21) employed an identical approach for malunited lateral and posterior malleolar fractures, confirming its efficacy in facilitating articular visualization, reduction, and fixation. Theoretically, this osteotomy provides direct visual access for the reduction and fixation of talar body fractures; however, no prior studies have documented its clinical application in this context. Inspired by Li (6), the authors implemented this osteotomy technique for Sneppen type I, II, and V talar body fractures. Combined medial and lateral approaches have been used to treat type V fractures. The procedure was technically straightforward intraoperatively, yielded stable fixation, and produced satisfactory postoperative outcomes.

#### Advantages over conventional osteotomies

A low osteotomy level preserves the distal tibiofibular syndesmosis, eliminating the need for early syndesmotic screw removal. Large osteotomy fragments with extensive oblique inter-fragmentary contact enhance fixation stability and minimize the risk of hardware failure and nonunion. Superior articular assessment, particularly valuable in concomitant distal tibial fractures, enables the direct evaluation of articular reduction and tibiotalar congruence, a capability unmatched by alternative approaches. Reduced iatrogenic injury: Intraoperative findings revealed preexisting ATFL rupture or avulsion fractures in four cases, which paradoxically facilitated surgical exposure. ATFL reconstruction was augmented using 3.5-mm metallic suture anchors coupled with a modified Broström technique to prevent postoperative instability. This reliance on ATFL repair means that early ankle stability depends on soft-tissue healing, and the extensive lateral soft-tissue injury and dissection needed for exposure may lengthen the rehabilitation course. This factor, together with the intra-articular injury, the three-month period of restricted weight bearing and subsequent capsular scarring, likely contributes to the marked restriction in ankle range of motion observed at follow-up. These considerations also support a cautious interpretation of the functional outcomes, which are probably best regarded as an acceptable salvage result for this severe injury pattern rather than a full functional recovery.

#### Technical considerations

Supplemental incisions are warranted for concomitant distal tibial fractures to mitigate excessive soft tissue tension and compromise wound healing. The superficial peroneal nerve requires meticulous protection. Articular surfaces must be handled with extreme delicacy to avoid iatrogenic chondral injury. Osteotomy lines were demarcated using methylene blue. A 2.0-mm Kirschner wire may be inserted laterally into the talar body to stabilize the distal fragment and optimize exposure. Uniform application of compressive dressing and vigilant monitoring of the distal limb perfusion are imperative postoperatively.

### Limitations

This study has several limitations. The sample size of five patients limits the statistical power and generalizability of the findings. However, combined talar body and tibial pilon fractures are rare injuries, and patient accrual at a single center is inevitably constrained; given the paucity of literature on this injury combination, even small case series provide useful preliminary evidence. The single-arm design without a comparison group does not permit a direct assessment of the relative efficacy of the modified osteotomy against the established techniques discussed above. The advantages attributed to the current technique are therefore based on descriptive comparison with published data rather than controlled analysis. Although paired comparisons of range of motion were performed between the affected and contralateral ankles, the clinical outcomes—including the AOFAS scores, VAS scores, and time to Hawkins’ sign—should be interpreted with caution given the small cohort. All postoperative assessments were conducted by the treating surgical team without blinded or independent evaluation, which may introduce assessment bias. The mean follow-up of 26.6 months may not be sufficient to detect late complications such as progressive post-traumatic arthritis or talar avascular necrosis. The cohort was also skewed by sex, which may limit how well it represents the broader population that sustains these high-energy injuries. In addition, with only five patients the power of any formal statistical test is very limited; the paired range-of-motion comparisons were therefore analyzed using non-parametric methods and are best read as descriptive rather than confirmatory. Multicenter comparative studies with larger cohorts, longer follow-up, and independent outcome evaluation are needed to validate these preliminary findings.

## Conclusion

Talar body fractures with tibial pilon fractures are rare and surgically challenging injuries with a high risk of ankle osteoarthritis and avascular necrosis. The modified lateral malleolar osteotomy approach is technically straightforward, provides excellent exposure to the tibiotalar joint surface and fracture fragments, facilitates reliable fixation, and yields acceptable short-term outcomes. This technique may be a viable alternative for isolated talar body fractures, comminuted fractures requiring bimalleolar osteotomy, and pilon fractures.

## Data Availability

The original contributions presented in the study are included in the article/Supplementary Material, further inquiries can be directed to the corresponding author.
